# Case Report: A New Tool for Anterior Chest Wall Reconstruction After Sternal Resection for Primary Or Secondary Tumors

**DOI:** 10.3389/fsurg.2021.691945

**Published:** 2021-07-20

**Authors:** Duilio Divisi, Davide Tosi, Gino Zaccagna, Andrea De Vico, Cristina Diotti, Roberto Crisci

**Affiliations:** ^1^University of L'Aquila, Department of MeSVA, Thoracic Surgery Unit, “Giuseppe Mazzini” Hospital, Teramo, Italy; ^2^Thoracic Surgery and Lung Transplant Unit, Fondazione Ca' Granda Ospedale Maggiore Policlinico, University of Milan, Milan, Italy

**Keywords:** thymoma, Ewing's sarcoma, sternectomy, sternal resection and reconstruction, titanium mesh

## Abstract

Sternal resection and anterior chest wall reconstruction techniques for malignant processes are not always standardized. We report an innovative method of sternal osteosynthesis in two patients, 65-year-old and 41-year-old women, with Ewing's sarcoma, and infiltrating thymoma, respectively. The first case manifested itself as a voluminous palpable mass while the second case was characterized for a paramediastinal mass widely extended to the anterior chest wall. Reconstruction with titanium mesh allowed the quick restoration of parietal stability, facilitating respiratory dynamic and recovery of patients.

## Introduction

Ewing's Sarcoma (ES) is the second most frequent primary bone cancer in adolescents and is rarely found after the age of 30. It mainly affects the diaphyses of the long bones and, in only one-third of cases, the soft tissues. It probably originates from neural crest or mesenchymal stem cells and shows three variants of histology: classical ES (the most common), primitive neuroectodermal tumor (PNET) and atypical ES. Classical ES is genetically characterized by a translocation involving the EWSR1 gene (locus22q12) and the ETS transcription factors with the formation of an aberrant chimeric protein (EWSR1-ETS) that promotes oncogenesis (1, 2). Thymoma is the most common primary anterior mediastinal neoplasm and generally affects adults with the ages between 40 and 70. It is characterized by a higher survival rate than thymic carcinoma (respectively, 78 and 40% at 5 years). It is associated with myasthenia gravis in a percentage between 30 and 50% and, unlike thymic carcinoma, thymoma spreads with greater difficulty; therefore, surgery is the treatment of choice (3, 4). In the literature many techniques have been proposed for the reconstruction of the sternum. The goal is to re-establish the continuity of the anterior chest wall trying to limit the negative effects on the respiratory mechanics. If the defect is <5 cm, the stabilization can be carried out by rigid osteosynthesis devices without reduction of the elastic properties of the thorax. Beyond this threshold and in case of considerable loss of substance, other methods for reconstruction must be considered (5). The autologous tissue is no longer widely used due to the high risk of infection (6). The heterologous prosthesis (PTFE, silicone, carbon fiber, methyl methacrylate) or bioprosthetics (human acellular dermal matrixes, Surgisis, Permacol) show low risk of infection and easy adaptability but the lack of intrinsic stiffness does not allow an adequate protection of the underlying vital structures (7, 8). The rigid devices such as titanium plates have proven to result in improved chest wall stability (9); unfortunately, their use alone can compromise the dynamics of the thorax especially in the total sternectomy for malignant disease. We describe the reconstruction of the anterior chest wall after sternal resection with a new rigid titanium mesh, able to stabilize the rib cage without support of additional autogenous tissue or prosthetic material.

## Case 1

### Patient Information

Sixty-five year old female with palpable mass of the sternum. Lesion showed volumetric progression (doubling in volume in the last month was reported), causing anterior chest pain refractory to painkilling treatment.

### Clinical Findings and Diagnostic Assessment

Computed tomography (CT) of the thorax revealed a lesion in the sternal body (5 × 4cm), involving the manubrium and the body of the sternum and the internal mammary vessels (Figures 2A,B). Considering the time elapsed between the patient's awareness of the mass and the first access to the hospital (3 months) and based on the rapid volumetric increase, the multidisciplinary team suggested surgery.

### Therapeutic Intervention

**Figure 1:**
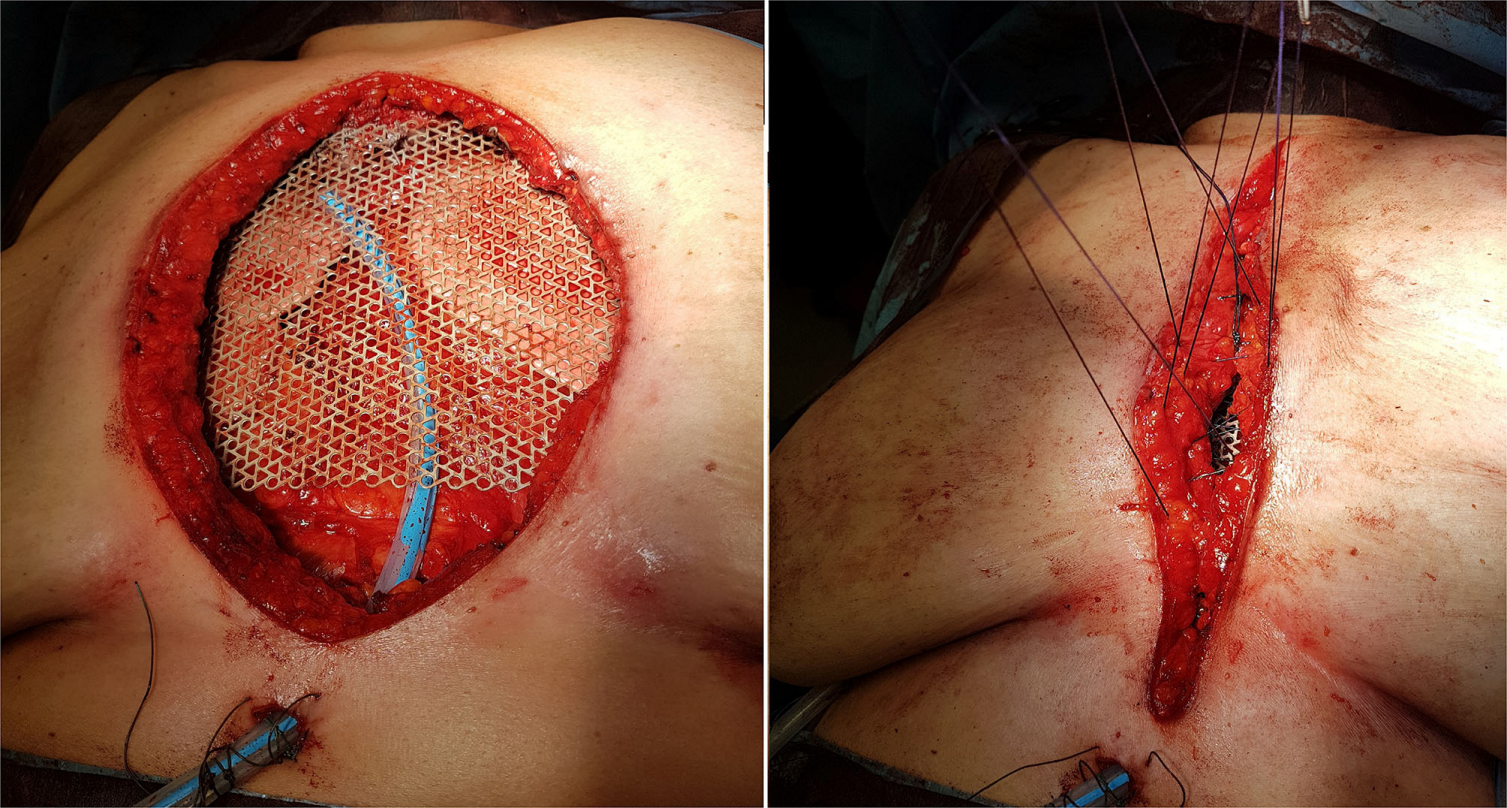
Shaped titanium mesh that entirely covers the anterior wall defect.

After longitudinal skin incision in the midline of the chest and dissection of pectoralis major muscle in the midline sternal insertions, an intraoperative biopsy was performed which revealed a sarcoma. Ribs were resected at level of the chondro-sternal cartilages (from the second to the seventh bilaterally) and the sternum was removed with the chondro-sternal joints “en bloc.” The reconstruction of the anterior chest wall was carried out with a titanium mesh, modeled according to the characteristics of the thorax. The device was fixed by non-absorbable polyfilament stitches inserted between the mesh and the anterior arc of the ribs. The implant was strengthened through myoplasty with pectoral major superiorly and rectus abdominis inferiorly (Figure 1).

### Outcomes

**Figure 2:**
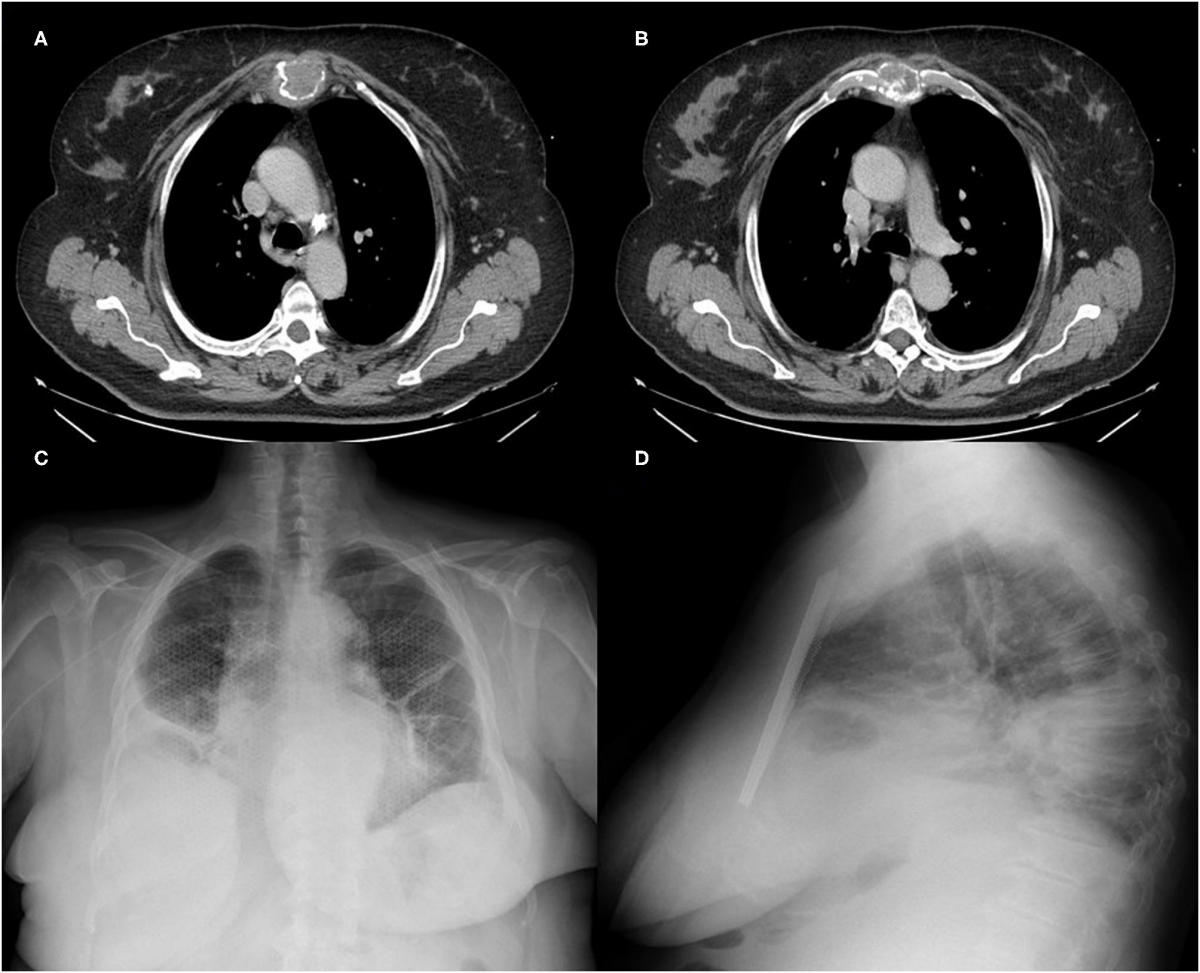
**(A,B)** Preoperative CT of the thorax showed a wide destruction of the upper 2/3 of the sternum by the neoplastic mass; **(C,D)** Chest X-ray at 40 days displayed the correct positioning of the titanium mesh, perfectly integrated into the wall.

No postoperative complication was noted. Patient was discharged 8 days after surgery and the definitive histological examination showed the classical Ewing's Sarcoma. The 40-day follow-up revealed the perfect mesh tightness with no signs of displacement (Figures 2C,D) and paradoxical movement of the chest. The preservation of the elasticity and the dynamics of the thorax, avoiding respiratory failure, was highlighted by the clinical-radiological findings and by blood-gas parameters (partial pressure of oxygen: 97.56 mmHg; carbon-dioxide levels: 31.00 mmHg; oxygen-blood saturation: 98.76%).

## Case 2

### Patient Information

The patient is a 41 year old female, affected by myasthenia gravis, positive for anti-acetylcholine receptor (anti-AChR) antibodies and under medical treatment. She underwent thymectomy by median sternotomy in the 2 two thousand and10 associated with “en bloc” subtotal resection of pericardium, replaced with a polytetrafluoroethylene mesh. Histological examination showed a thymoma B2, with infiltration of the thymic capsule and the mediastinal adipose tissue. Patient underwent adjuvant radiotherapy on the thymic lodge, with a total dose of 50 Gy, and the oncologic follow-up was negative until 2016.

### Clinical Findings and Diagnostic Assessment

**Figure 3:**
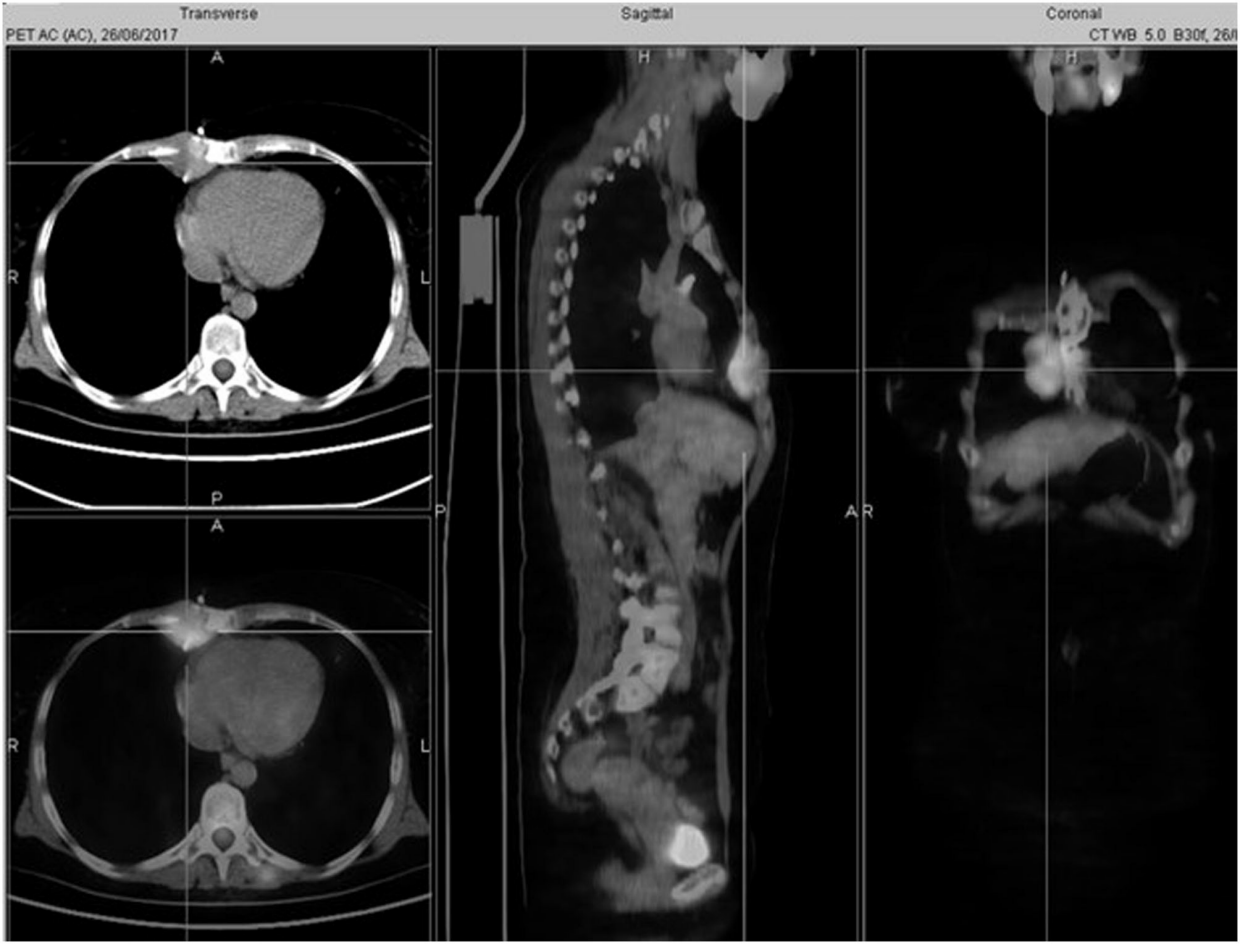
Preoperative PET/CT displayed a right paramediastinal mass infiltrating the sternal body.

Computed tomography (CT) of the thorax carried out in June 2017, due to cough and retrosternal weight, revealed a paramediastinal mass (6 × 4 × 2 cm), largely infiltrating sternal body, chondro-sternal joints, xiphoid process, and pericardial fat. Positron emission tomography/computed tomography (PET/CT, Figure 3) displayed a standardized uptake value (SUV) max of 6.5 only at site of the lesion. After a multidisciplinary evaluation, surgery was proposed as the first approach.

### Therapeutic Intervention

**Figure 4:**
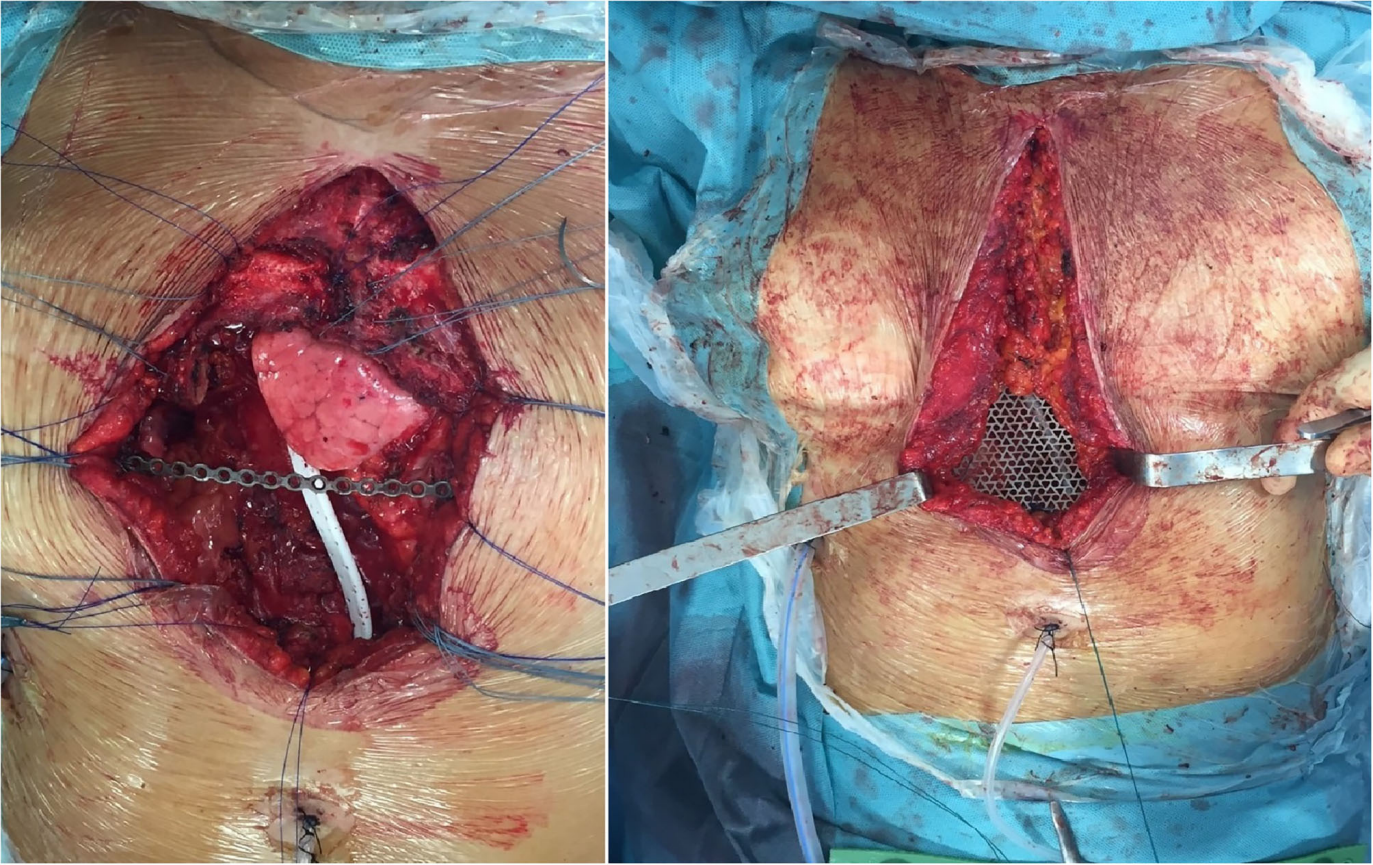
Titanium mesh resting on a titanium support plate.

The patient was positioned supine. A vertical skin incision was performed. Ribs from the third to the seventh were bilaterally resected with a rib cutter. The sternal body was transversally divided with an oscillating saw. We revealed a neoplastic infiltration of the right upper pulmonary lobe. Therefore, a wedge resection by endostapler was performed in order to obtain “en bloc” tumor resection, including the sternal body with the chondro-sternal joints, lung and pericardial fat. The bony chest wall was stabilized using a 4 mm thick fenestrated titanium mesh (original size 200 × 150 mm), which was fixed to the ribs by prolene stitches and reinforced with a titanium bar in the central part of the prosthesis (Figure 4). Pectoralis muscles were mobilized in order to cover the chest defect, before suturing.

### Outcomes

The patient was transferred to the intensive care unit under mechanical ventilation, and was extubated on postoperative day 1. No paradoxical movements were revealed, chest wall stability was assessed both in supine and seated position, and respiratory physiotherapy exercises were started. The postoperative course was free from respiratory complications. Histological examination confirmed a relapse of thymoma B2 (pT3Nx). A CT-scan 6 months after surgery confirmed that the titanium mesh was correctly in place. The patient complained of chest pain in the right hemithorax for some months, but no breathlessness.

## Discussion

In case of anterior chest wall tumors involving bones, muscles and soft tissues, surgical resection with wide-free margins is the most relevant prognostic factor for disease-free survival, and often massive demolitions are required to obtain a radical resection (10). The sternochondral replacement approach is not yet standardized. In fact, the choice of the best strategy is decided from case to case based on the experience of surgeon. Many techniques have been proposed over the years (11–13) in order to restore the integrity of the anterior chest wall and to prevent the organs injury, maintaining the physiological and functional characteristics of the thorax. Myocutaneous flaps or prosthetic meshes do not sufficiently protect the organs from external trauma and can accentuate the deformity of the chest. The bone autograft or allograft is strongly compromised by the tendency to develop infections and the low availability of donors, respectively. An innovative technique of sternal transplantation for anterior chest wall reconstruction has been reported by Dell'Amore et al. (14) A tailored sternal allograft is fixed to the patient's chest wall using titamium bars and is covered with a pectoralis muscle flap. Even if its use is very limited, this technique provides excellent functional and cosmetic results. The custom-made implant seem to be a valid alternative but is not easily accessible and widely diffused both for the technical and economic aspects, although the problem of costs is not carefully analyzed in the literature. Titanium plates in anterior chest wall reconstruction after total sternectomy are solely used as rigid support for the placement of a mesh. In fact, applying this method alone patient could be exposed to postoperative flail chest and thoracic organs herniation or injuries (15–18). One of the most effective reconstructive techniques after total sternectomy is the “sandwich” method using methyl methacrylate wrapped between two layers of polypropilene or marlex mesh. This approach is associated with optimal functional results and the encapsulation of methyl methacrylate reduces the risk of dislocation, even in case of rupture. In addition, the “sandwich” is well-anchored to the chest wall using stainless-steel sutures, which decreases even more the risk of displacement (10). Among other available surgical strategies, the original “rib-like” technique proposed by Girotti et al. (19) reproduces the best possible anatomy. Authors used an aluminum cast covered by a non-absorbable mesh and filled over the mesh with radiopaque acrylic resine to build a tailored semi-rigid prosthesis. The surgical implant is then anchored to the costal stumps, improving the biocompatibility of chest wall prostheses. However, the choice of the proper material and of the most adequate reconstruction technique depends on the size and the location of the defect, the age and the physical characteristics of the patient, the expertise of the surgeon. We experienced the titanium mesh (size 200 × 140 mm and thickness 0.4–0.6 mm), strengthened by myoplasty with pectoralis major and abdominal muscles, for the following reasons: (a) titanium is an inert element with a low risk of infection. The special production technique (Photochemical Etching) eliminates thermal risks due to cutting with other methods, thus preserving the metallurgical properties of titanium and providing constant performance of device during and after surgery; (b) the mesh architecture prevents the accumulation of fluids. The particular design at triangle gives it indeformability and mechanical resistance with an excellent elasticity. Hence, this mesh can be fixed both to the bone (with screws and/or not resorbable suture threads) and to tissues (with not resorbable suture threads); (c) the semi-rigid structure allows to shape the prosthetic material on the basis of the specific anatomy of the thorax. These peculiarities lead the subsequent fibrosis to engulf the implant in the chest wall, solidifying it to the myoplasty and making a further rigid support, as we have practiced in the second patient, not mandatory. Therefore, the minimal reduction of elasticity is balanced by the parietal strength improvement, the restoration of the physiological ventilatory mechanics and excellent aesthetic findings. Furthermore, the considerable economic savings must be considered. In fact, the cost of the mesh is 3,300 € and is lower than using 4 rigid titanium plates (1,400 € for single device) and a soft mesh (from 2,747 € for a 26 × 34 cm in polytetrafluoroethylene to 8,400 € for the one in porcine dermal collagen) normally required for the synthesis of the anterior chest wall. In conclusion, the use of titanium mesh has proved to be a feasible, safe and effective technique, always customizing it according to the characteristics of the patients.

## Data Availability Statement

The original contributions presented in the study are included in the article/supplementary material, further inquiries can be directed to the corresponding author/s.

## Ethics Statement

Written informed consent was obtained from the patients for the publication of any potentially identifiable images or data included in this article.

## Author Contributions

DD contributed to conception and design of the study. DD and DT wrote the first draft of the manuscript. AD, GZ, CD, and RC wrote sections of the manuscript. All authors contributed to manuscript revision, read, and approved the submitted version.

## Conflict of Interest

The authors declare that the research was conducted in the absence of any commercial or financial relationships that could be construed as a potential conflict of interest.
